# Rheological Properties of Cholesteric Liquid Crystal with Visible Reflection from an Etherified Hydroxypropyl Cellulose Derivative

**DOI:** 10.3390/polym14102059

**Published:** 2022-05-18

**Authors:** Kazuma Matsumoto, Yuki Ogiwara, Naoto Iwata, Seiichi Furumi

**Affiliations:** Department of Chemistry, Graduate School of Science, Tokyo University of Science, 1–3 Kagurazaka, Shinjuku, Tokyo 162-8601, Japan; 1322628@ed.tus.ac.jp (K.M.); 1321523@ed.tus.ac.jp (Y.O.)

**Keywords:** cellulose, cholesteric liquid crystal, light reflection, rheology

## Abstract

Optical properties of hydroxypropyl cellulose (HPC) derivatives have been widely investigated for their ability to exhibit cholesteric liquid crystal (CLC) phase. However, there are only a limited number of studies on their rheological properties even though they are quite important for the applications of such HPC derivatives to the versatile CLC photonic devices. In this article, we report on the optical and rheological properties of an HPC derivative possessing pentyl ether groups in the side-chains. The etherified HPC derivative exhibited thermotropic CLC phase with light reflection in the temperature range between 25 °C and 120 °C. After the HPC derivative was heated once at isotropic phase, followed by being cooled to the CLC phase, the reflection peak could not be observed, even at the CLC phase. At this stage, the HPC derivative exhibited solid-like rheological responses compared to that of sheared at a constant shear rate of 1.0 s^−1^. Such differences in the optical and rheological properties of the HPC derivative can be ascribed to the difference in CLC orientation state. From the rheological results, the etherified HPC derivative showed liquid-like behavior rather than the esterified HPC derivatives. This evidence provides a promising clue for fabricating high-quality CLC devices by the facile CLC orientation.

## 1. Introduction

**Figure 1:**
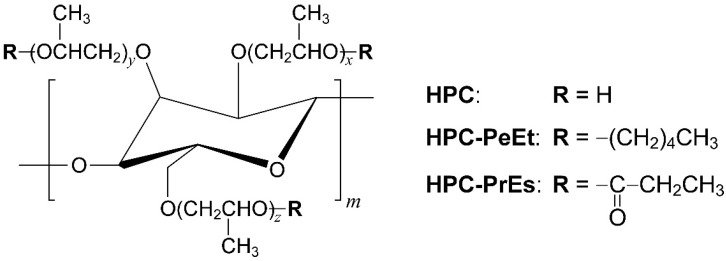
Chemical structures of pristine hydroxypropyl cellulose (HPC) and its etherified derivative possessing pentyl side-chains (HPC-PeEt). To compare the rheological results of HPC-PeEt in Figure 5, the previous results of an esterified HPC derivative possessing propionyl side-chains (HPC-PrEs) will be discussed in Section 3.4.

Cellulose is the most abundant natural polymer on the Earth. Cellulose derivatives are attractive because of their safety and biocompatibility. Hydroxypropyl cellulose (HPC), as depicted in Figure 1, is a cellulose derivative that can be obtained by reacting natural cellulose with propylene oxide. Owing to its safety, HPC has been utilized not only as food and pharmaceutical additives, but also as thickeners and coating agents in interdisciplinary industrial fields [1,2,3,4].

Another interesting property of HPC is its ability to exhibit cholesteric liquid crystal (CLC) phase with light reflection [5]. Gray and Werbowyj succeeded for the first time in the preparation of CLCs using pristine HPC. When powdery HPC is dissolved in water at high concentration, the viscous solutions show lyotropic CLC phase with visible reflection characteristics depending on the concentration [6,7]. Following their archetypal demonstrations, numerous studies have shown that HPC derivatives also exhibit thermotropic CLC phase with visible reflection after the hydroxy groups in side-chains of HPC are chemically substituted with alkyl chains through ester [8,9] or ether bonds [10,11,12], that is, esterified or etherified HPC derivatives, respectively. At CLC mesophase, the rod-shaped molecules spontaneously form the periodic helicoidal structure owing to their chirality, thereby enabling the emergence of unique light reflection at a specific wavelength. Such a light-reflection phenomenon can be recognized as a kind of Bragg reflection [13,14]. The maximum reflection wavelength (*λ*) can be numerically expressed according to the following equation proposed by De Vries [15]:

(1) λ=n p

where *n* stands for the average refractive index and *p* is the helical pitch length of CLC. In the case of thermotropic CLCs, the *p* value readily fluctuates with temperature. As a result, the reflection colors of CLCs are significantly dependent on the temperature, leading to their wide range of potential applications as temperature indicators, reflective color displays, full-color recording media, tunable lasers, and so forth [16].

In this context, the rheological properties of CLCs have been attracting interest for decades [17,18]. To understand their rheological properties, which are distinct from those of other polymer solutions, aqueous solutions of cellulose derivatives have been one of the most frequently investigated motifs. This is because the cellulose derivatives generally form the rigid helical conformation in solution [19]. Based on this background, a few precedents have been made on the rheological behavior of lyotropic CLCs from mixtures of cellulose derivatives such as HPC or sulfonated cellulose nanocrystal with water [20,21].

In the case of thermotropic CLCs, it is quite interesting that sticky products can be obtained by the simple substitution of alkyl chains to the hydroxy groups of pristine HPC. This phenomenon can be observed when HPC is either esterified [22] or etherified [14]. In addition, it is of prime importance to understand the rheological behavior of CLCs from HPC derivatives because they are potentially applicable to CLC photonic devices with eco-friendliness for the next-generation sustainable society. To date, several previous studies have dealt with the CLCs of esterified HPC derivatives possessing alkanoyl side-chains. For instance, our previous report has shown that the rheological properties of esterified HPC derivatives are greatly affected by the CLC orientation [23,24]. On the other hand, we have recently established a promising methodology to synthesize etherified HPC derivatives possessing alkoxy side-chains [11]. Such etherified HPC derivatives also exhibit thermotropic CLC phase in a manner similar to that of esterified HPC derivatives. Furthermore, it is predictable that the completely etherified HPC derivatives exhibit stable reflection colors under highly humid conditions because of the chemical stability of the ether linkage rather than the ester [11]. Such stability of the etherified HPC derivatives is highly attractive for the technological developments of CLC photonic devices when compared to the esterified HPC derivatives whose optical and rheological properties have been extensively studied. However, there has been no report to explore the rheological behavior of etherified HPC derivatives.

In this research, we investigated the optical and rheological properties of an HPC derivative possessing pentyl side-chains through ether bonds (HPC-PeEt), as shown in Figure 1. The transmission spectral measurements revealed that a reflection peak of HPC-PeEt is reversibly shifted in the full visible-wavelength range by changing the temperature between 25 °C and 120 °C, arising from the thermotropic CLC feature. The rheological behavior of HPC-PeEt was greatly dependent on the CLC orientation state, similar to the case of esterified HPC derivatives. This report will contribute to the detailed comprehension of the correlation between CLC and rheological properties for various cellulose materials.

## 2. Experimental Section

HPC (viscosity of 2.0 wt% aqueous solution: 2.0–2.9 mPa·s) as the starting material was purchased from FUJIFILM Wako Pure Chemical Co (Osaka, Japan). The number average molecular weight (*M*_n_) and weight average molecular weight (*M*_w_) were 2.68 × 10^4^ and 6.37 × 10^4^, respectively, as determined by measurement of size exclusion chromatography (SEC) equipped with a refractive index detector (RI-4030 and PU-4180, JASCO, Tokyo, Japan) calibrated using the polystyrene standards. In the SEC measurements, tetrahydrofuran (THF) was used as the eluent. HPC was dried in vacuo overnight before use. When the ^1^H-NMR spectrum of this pristine HPC was measured in CDCl_3_, the molar amount of chemically combined propylene oxide per anhydroglucose unit, that is, the molar substitution (*MS*), was found to be 3.98. Therefore, the average molecular weight per anhydroglucose monomer unit could be calculated to be 394. Subsequently, the changes in ^1^H-NMR spectrum of pristine HPC were evaluated during titration with trichloroacetyl isocyanate. From the ^1^H-NMR spectral changes, the number of hydroxy groups substituted per anhydroglucose unit, that is, the degree of substitution (*DS*) was estimated to be 2.40, according to the previous report [25]. Dehydrated *N*-methyl-2-pyrrolidone (NMP) was obtained from Kanto Chemical Co., Inc. (Tokyo, Japan). 1-Bromopentane (PeBr) was purchased from Tokyo Chemical Industry (Tokyo, Japan). Sodium hydroxide and potassium iodide were acquired from FUJIFILM Wako Pure Chemical Co. These reagents were used as received.

HPC-PeEt was synthesized by Williamson ether synthesis as reported in our previous study [11]. First, 5.00 g of HPC was completely dissolved in 80.0 mL of dehydrated NMP, and was subsequently added by 23.6 mL of PeBr (5.00 eq. to hydroxy groups of HPC). After stirring for 30 min at 65 °C, 7.60 g of powdered sodium hydroxide (5.00 eq. to hydroxy groups of HPC) and 1.58 g of potassium iodide (5.00 mol% to PeBr) were added into the reaction solution. After that, this reaction mixture was stirred at 65 °C for 48 h. Then, the reaction mixture was purified by two rounds of centrifugation at 1.0 × 10^4^ rpm for 5 min to remove any sediment such as sodium bromide. PeBr was evaporated from the supernatant by heating at 60 °C in vacuo for 30 min. The residue was dialyzed against an equivolume mixture of methanol and water for 3 h, and the dialysis was continued for additional 48 h in an equivolume mixture of THF and methanol. The product was obtained by reprecipitation to a mixture of water and methanol at the volume ratio of 9:1, respectively, and was finally freeze-dried to afford the purified HPC-PeEt. The characterization of HPC-PeEt was carried out by FT-IR spectroscopy using an attenuated total-reflection module (FT-IR4700 and ATR PRO ONE, JASCO) and ^1^H-NMR spectroscopy (JNM-ECZ400S, JEOL, Tokyo, Japan) for the molecular structure as well as SEC analysis for the *M*_n_ and *M*_w_ values.

In order to measure the optical properties, a CLC cell was fabricated by sandwiching HPC-PeEt between a pair of glass substrates. The cell gap was adjusted by polytetrafluoroethylene film spacers to a thickness of ~200 µm. Transmission spectra of the CLC cell were taken on a compact charge-coupled device (CCD) spectrometer (USB2000+, Ocean Optics, FL, USA) equipped with an optical fiber. The CLC cell was illuminated with white light from a tungsten halogen light source (Ocean Optics, HL2000). The collinearly transmitted light from the CLC cell was focused through two pieces of achromatic doublet lenses and collected into the entrance of optical fiber connected with the CCD spectrometer. The temperature of the CLC cell was precisely controlled using a hot-stage system (HS82 and HS1, Mettler Toledo, OH, USA).

The viscosity measurements were conducted using a stress-controlled rheometer (MCR102, Anton Paar, Graz, Austria) equipped with a stainless-steel parallel plate with a diameter of 25 mm and a forced convection oven (Anton Paar, CTD450) for controlling the temperature. In the measurements, HPC-PeEt was sandwiched at a gap of ~500 µm. Angular frequency (*ω*) dependence of HPC-PeEt was analyzed using the above-mentioned rheometer. Both the storage modulus (*G*′) and loss modulus (*G″*) were measured at the range of *ω* between 0.1 rad/s and 100 rad/s at 25 °C. The strain amplitude was adjusted in the range between 0.2% and 1.2%, which is small enough to measure the linear viscoelasticity. Prior to this measurement, HPC-PeEt was either pre-heated or pre-sheared in order to erase any orientational history of CLC structures. As the pre-heat treatment, HPC-PeEt was heated at 155 °C for 10 min in the rheometer. This temperature was higher than the isotropic phase transition temperature of HPC-PeEt, corresponding to 150 °C, to retain the random orientation of cholesteric texture as confirmed by the polarized optical microscopic observation. On the other hand, the pre-shear treatment was conducted by shearing HPC-PeEt at a constant shear rate of 1.0 s^−1^ for 5 min at 25 °C, followed by leaving it to stand for 7 min at the same temperature after stopping the shearing force.

## 3. Results and Discussion

### 3.1. Characterization of HPC-PeEt

**Figure 2:**
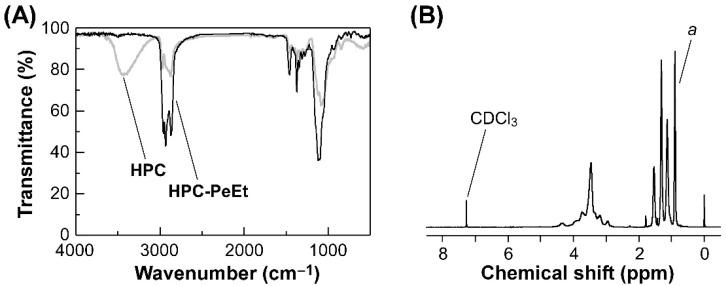
(**A**) FT-IR spectra of pristine HPC (gray line) and HPC-PeEt (black line). (**B**) ^1^H-NMR spectrum of HPC-PeEt in CDCl_3_. The peak *a* is assigned to terminal methyl groups in the pentyl ether side-chains of HPC-PeEt.

First, we measured FT-IR and ^1^H-NMR spectra to confirm that all hydroxy groups of pristine HPC are substituted with pentyl ether side-chains after etherification. Figure 2A,B show the FT-IR spectra of pristine HPC and HPC-PeEt and ^1^H-NMR spectrum of HPC-PeEt, respectively.

The FT-IR spectral results are shown in Figure 2A. The pristine HPC exhibited a broad peak in the wavenumber range from 3000 cm^−1^ to 3600 cm^−1^, which is assigned to the stretching vibration of hydroxy groups. In contrast, this peak disappeared in the FT-IR spectrum of HPC-PeEt, suggesting that the hydroxy groups of pristine HPC are almost substituted with pentyl ether groups [11]. The etherification of HPC was also confirmed from the ^1^H-NMR spectral results of HPC-PeEt [11]. Figure 2B represents the ^1^H-NMR spectrum of HPC-PeEt in CDCl_3_ [25]. A sharp peak at 0.99 ppm can be assigned to the terminal methyl groups in the pentyl ether groups of HPC-PeEt. As the quantitative analysis, the etherification degree (*DS*_Pe_) in a monomer unit of HPC was calculated by the following equation:

(2) $$DS_{\mathrm{Pe}}=\frac{A\left(7+6MS\right)}{3W-11A}$$

where *A* is the integrated value of the peak *a* in Figure 2B and *W* is the sum of the integrated values of all protons in HPC-PeEt. The mathematical derivation of Equation (2) is available in the Appendix A, as described below. As mentioned in the Experimental Section, *MS* was determined to be 3.98. It should be noted that the maximal *DS*_Pe_ value is 3.00 since the monomer unit of HPC has three hydroxy groups, as depicted in Figure 1. By applying the experimental results to Equation (2), the *DS*_Pe_ value of HPC-PeEt was estimated to be 3.00. This result indicated that all hydroxy groups of pristine HPC are completely substituted with pentyl side-chains through the ether bonds. Moreover, the values of *M*_n_ and *M*_w_ of pristine HPC and HPC-PeEt were evaluated by the SEC analysis. As compiled in Table 1, both *M*_n_ and *M*_w_ of HPC-PeEt were in the same order of magnitude as those of pristine HPC, implying that no depolymerization in the main-chain of HPC might occur in the etherification process.

Previously, Greiner and colleagues have revealed that the reflection peak wavelength of CLC from the esterified HPC derivatives is greatly affected by the esterification degree, that is, the amount of remaining hydroxy groups of the HPC backbone [26]. In our preliminary experiment, such tendency of reflection property was also observed for the etherified HPC derivatives. In addition, the rheological properties of HPC derivatives might be vulnerable to the remaining hydroxy groups of the HPC backbone due to the formation of hydrogen bonds. Therefore, we anticipated that HPC-PeEt synthesized in this study is suitable for investigating the optical and rheological properties for the CLCs of completely etherified HPC derivatives.

### 3.2. Optical Properties of HPC-PeEt

**Figure 3:**
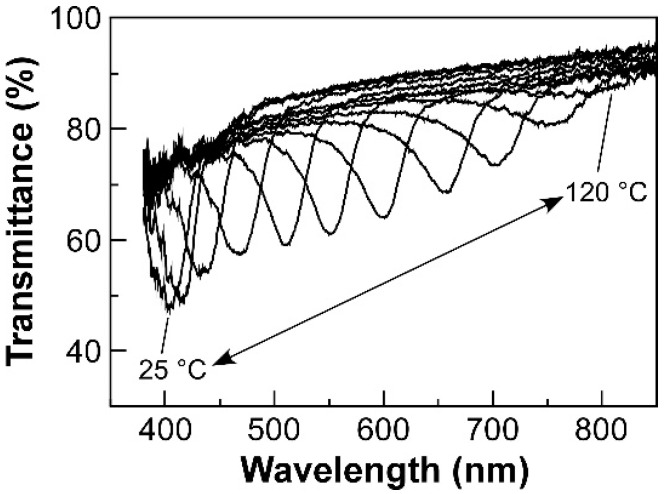
Changes in the transmission spectrum of the CLC cell of HPC-PeEt upon heating process from 25 °C to 120 °C.

HPC-PeEt exhibited thermotropic CLC phase with visible reflection characteristic. Figure 3 shows changes in the transmission spectrum of a CLC cell comprising HPC-PeEt upon heating process. When the temperature of the CLC cell was adjusted to 25 °C, a reflection peak appeared at 400 nm, corresponding to the blue reflection color. Subsequently, when the temperature was gradually elevated from 30 °C to 120 °C at the intervals of 10 °C, the reflection peak was continuously red-shifted to the near-infrared region of 780 nm. This can be attributed to the increase in *p* value of CLC as the temperature rises [27]. Successively, the reflection peak was blue-shifted from 780 nm to 400 nm upon cooling from 120 °C to 25 °C. Such a reversible change in reflection peak by temperature is the typical characteristic of thermotropic CLC phase.

These spectral results were good agreement with our previous report on the thermotropic CLC from an HPC derivative completely substituted with pentyl ether groups [11]. In addition, such a wide wavelength shift in reflection peak of CLC, which fully covers the visible wavelength region as well as near infrared region, is in high demand for the technological applications of CLC photonic devices. Considering that the rheological behavior of CLC is greatly affected by the difference of CLC helical structure, it is necessary to choose an etherified HPC derivative with a similar reflection property to that of esterified HPC derivatives for fair comparison of their rheological properties. As another advantage, HPC-PeEt exhibited thermal stability in its reflection properties because there was no distinct wavelength shift in the reflection peak even after heating at 120 °C for 5 h, indicating no thermally-induced decomposition of HPC-PeEt. For these reasons, we concluded that HPC-PeEt is suitable for investigating the rheological properties of cellulose-based thermotropic CLCs similar to the esterified HPC derivatives reported in our previous studies [23,24].

### 3.3. Pre-Treatments of HPC-PeEt before Rheological Measurements

For fair evaluation of the rheological properties of CLCs, it is essential to control the molecular orientation states at the CLC phase. This is because the rheological properties are greatly affected by the internal molecular structures [23,24]. To overcome this problem, the pre-heat and pre-shear treatment defined in the Experimental Section might be useful for the rheological measurements, to ensure the disordered and aligned states, respectively, of molecular structure at the CLC phase.

**Figure 4:**
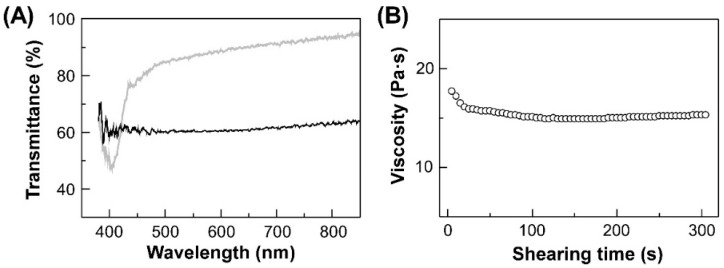
(**A**) Transmission spectra of the cell of HPC-PeEt before (gray line) and after (black line) the pre-heat treatment. (**B**) Changes in viscosity of HPC-PeEt as a function of shearing time. This measurement was performed at the constant shear rate of 1.0 s^−1^ and at 25 °C.

The pre-heat treatment was conducted by heating HPC-PeEt at 155 °C for 10 min in advance. This condition was determined from both transmission spectral measurements and polarized optical microscopic observation of the CLC cell as follows: Figure 4A shows the transmission spectra of the CLC cell of HPC-PeEt measured at 25 °C before and after the pre-heat treatment. As mentioned in Section 3.2, the CLC cell showed a reflection peak at 400 nm as blue color before the pre-heat treatment (Figure 4A, gray line). When this CLC cell was observed by a polarized optical microscope under crossed Nicols while heating process, no transmission light was observed at 150 °C. This result indicated that the birefringence of HPC-PeEt completely disappears due to the thermally-induced phase transition from CLC to isotropic. Thus, the CLC structure is assumed to be the disordered state at this temperature. After the temperature was gradually lowered to 25 °C, the reflection peak could not be revived even though the CLC cell reflected blue light of 400 nm at the same temperature before heating (Figure 4A, black line). At this stage, the birefringence observed by the polarized optical microscope was weaker than before the heating treatment at 155 °C. These results inferred that the disordered CLC structure can be partially preserved even at CLC phase of 25 °C after the pre-heat treatment. This phenomenon can also be supported by the experimental results that the baselines in the transmission spectra decline from ~85% to ~60% after the pre-heat treatment, which can be ascribed to the light scattering caused by disordered CLC structure (Figure 4A).

The pre-shear treatment was conducted by shearing HPC-PeEt at a constant shear rate of 1.0 s^−1^ for 5 min at 25 °C. Figure 4B shows the changes in viscosity of HPC-PeEt as a function of shearing time. As evident from this experimental profile, the viscosity gradually decreased upon shearing, and became constant after 80 s reaching its steady state. Such decrease in viscosity can be ascribed to the molecular orientation of CLC by shear-flowing. This suggests that the shearing time needed for the stationary state of CLC orientation of HPC-PeEt is 80 s. Therefore, we determined that the pre-shearing for 5 min is long enough to orient the CLC structure of HPC-PeEt.

### 3.4. Rheological Properties of HPC-PeEt

**Figure 5:**
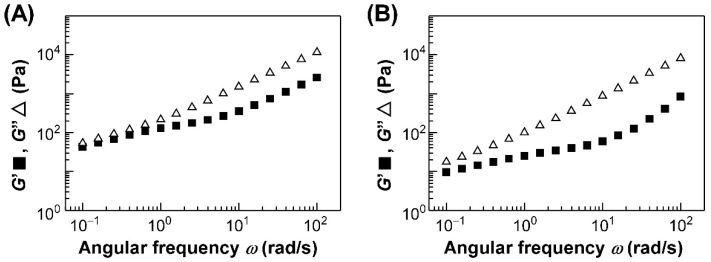
Angular frequency dependence of the storage modulus (*G*′; closed squares) and loss modulus (*G*″; open triangles) values of HPC-PeEt measured at 25 °C. Before the rheological measurements, HPC-PeEt was either pre-heated (**A**) or pre-sheared (**B**).

The rheological properties of HPC-PeEt were affected by the difference in the pretreatments of HPC-PeEt. Figure 5A,B show the angular frequency (*ω*) dependence of storage modulus (*G*′) and loss modulus (*G*″) of HPC-PeEt treated by pre-heating and pre-shearing, respectively. The measurements were performed at 25 °C. Although the rheological measurements of HPC-PeEt were repeated at least three times, we found that the reproducibility is sufficient.

In the case of pre-heated HPC-PeEt, the curves of *G*′ and *G*″ can be divided into two regions depending on the *G*′ and *G*″ values. In the *ω* range above 1.0 rad/s, *G*″ clearly exceeded *G*′, pronouncing that HPC-PeEt exhibits liquid-like behavior (Figure 5A). However, *G*′ and *G*″ were almost equal in the *ω* range below 1.0 rad/s, indicative of the solid-like behavior of HPC-PeEt in this *ω* range. In contrast to the pre-heat treatment, the curves of *G*′ and *G*″ for pre-sheared HPC-PeEt were distinct from those of pre-heated HPC-PeEt because *G*″ was constantly larger than *G*′ in the entire *ω* range between 10^−1^ rad/s and 10^2^ rad/s (Figure 5B). This difference in the relationship of *G*′ and *G*″ in the lower *ω* region, that is, the solid-like behavior of pre-heated HPC-PeEt, can be ascribed to the disordered state of CLC structure. This is also supported by both transmission spectral measurements and polarized optical microscopic observations, as noted in Section 3.3. Interestingly, HPC-PeEt treated by pre-heating and pre-shearing showed a similar *ω* dependence at the higher *ω* region. The slopes of *G*′ and *G*″ were estimated to be approximately 1.0 and 0.6, respectively. These values were much smaller than those of the ideal Newtonian fluid, which would be 2.0 and 1.0, respectively. Such smaller *ω* dependences of *G*′ and *G*″ can be attributed to the disturbed flow behavior of pre-heated and pre-sheared HPC-PeEt caused by the CLC structure.

Our previous studies have shown the rheological properties for the thermotropic CLC of an esterified HPC derivative possessing propionyl side-chains (HPC-PrEs) [23,24], as depicted in Figure 1. The rheological properties of both HPC-PeEt and HPC-PrEs can be characterized by the following two aspects. First, the *ω* dependence of *G*′ and *G*″ in the higher *ω* region was almost same since the *G*′ and *G*″ of HPC-PrEs became proportional to the *ω*^1.0^ and *ω*^0.8^, respectively. Second, the relationship between *G*′ and *G*″ in the lower *ω* region was dominated by the CLC molecular orientation state because it was dependent on the difference of the pre-treatment before the rheological measurements. This tendency was commonly observed for both HPC-PeEt and HPC-PrEs. However, the relationship of *G*′ and *G*″ in the lower *ω* region was slightly different, especially when measured after the pre-heat treatment. In the case of HPC-PrEs, *G*′ became larger than *G*″ in the lower *ω* region, while *G*′ and *G*″ were almost equal in the case of HPC-PeEt. Considering the fact that the birefringence of pre-heated HPC-PeEt is preserved after the pre-heat treatment, unlike HPC-PrEs, it can be assumed that the CLC of HPC-PeEt is more likely to be oriented than that of HPC-PrEs even after it was heated above its isotropic phase transition temperature. Such a quick orientation of CLC of HPC-PeEt is very attractive from the viewpoint of fabricating highly-ordered CLCs of HPC derivatives which are desired for the photonic devices with high performances.

## 4. Conclusions

In this report, we investigated the optical and rheological behavior of a thermotropic CLC of HPC-PeEt, that is, an HPC derivative tethering pentyl ether side-chains. HPC-PeEt exhibited thermotropic CLC phase with visible reflection property in the temperature range between 25 °C and 120 °C. The reflection peak wavelength could be reversibly shifted in the full visible-wavelength range from 400 nm to 780 nm upon heating and cooling processes. Moreover, the rheological properties of HPC-PeEt were found to be dependent on the molecular orientation states at the CLC phase. HPC-PeEt showed solid-like responses when heated above its isotropic phase-transition temperature, followed by cooling to 25 °C, even though it showed liquid-like behavior after shearing at a constant shear rate of 1.0 s^−1^. This is because the disordered CLC structures of HPC-PeEt prevent the flow behavior. These experimental results would be useful not only for understanding he fundamental physical properties of cellulose-based CLCs, but also for fabricating the environment-friendly CLC devices by utilizing cellulose derivatives for a sustainable society.

## Figures and Tables

**Table 1:** SEC results of pristine HPC and HPC-PeEt.

Sample	*M*_n_ (× 10^4^)	*M*_w_ (× 10^4^)	*M*_w_/*M*_n_
Pristine HPC	2.68	6.37	2.38
HPC-PeEt	1.65	4.89	2.97

## Data Availability

Data is contained within the article.

## Appendix A. Mathematical Derivation of Equation (2)

As addressed in Section 3.1, Equation (2) enables estimation of the *DS*_Pe_ value—the average number of the pentyl ether groups in a monomer unit of HPC-PeEt. We derived Equation (2) according to our previously reported procedure [28]. A peak of terminal methyl protons in the pentyl ether groups appeared around 0.99 ppm in the ^1^H-NMR spectrum of HPC-PeEt. Then, we defined the integrated value of this peak as *A* and the sum of intergrated values of the peaks derived from all protons of HPC-PeEt as *W*. Therefore, the *W* value obeyed Equation (A1) as follows:

(A1) $$W=7+6MS+11DS_{\mathrm{Pe}}$$

Because the *DS*_Pe_ value was calculated from the ratio of *A/3* and *W*, the relation was expressed as Equation (A2).

(A2) $$\frac{DS_{\mathrm{Pe}}}{7+6MS+11DS_{\mathrm{Pe}}}=\frac{A}{3W}$$

Lastly, Equation (A2) was rearranged so as to be Equation (2) relating to the *DS*_Pe_ value, as stated in Section 3.1.

## References

[1] Ali A., Ahmed S. (2018). Recent Advances in Edible Polymer Based Hydrogels as a Sustainable Alternative to Conventional Polymers. J. Agric. Food Chem..

[2] Kamita G., Frka-Petesic B., Allard A., Dargaud M., King K., Dumanli A.G., Vignolini S. (2016). Biocompatible and Sustainable Optical Strain Sensors for Large-Area Applications. Adv. Opt. Mater..

[3] Sarode A., Wang P., Cote C., Worthen D.R. (2013). Low-Viscosity Hydroxypropylcellulose (HPC) Grades SL and SSL: Versatile Pharmaceutical Polymers for Dissolution Enhancement, Controlled Release, and Pharmaceutical Processing. AAPS PharmSciTech.

[4] Chan C.L.C., Bay M.M., Jacucci G., Vadrucci R., Williams C.A., van de Kerkhof G.T., Parker R.M., Vynck K., Frka-Petesic B., Vignolini S. (2019). Visual Appearance of Chiral Nematic Cellulose-Based Photonic Films: Angular and Polarization Independent Color Response with a Twist. Adv. Mater..

[5] Almeida A.P.C., Canejo J.P., Fernandes S.N., Echeverria C., Almeida P.L., Godinho M.H. (2018). Cellulose-Based Biomimetics and their Applications. Adv. Mater..

[6] Werbowyj R.S., Gray D.G. (1976). Liquid Crystalline Structure in Aqueous Hydroxypropyl Cellulose Solutions. Mol. Cryst. Liq. Cryst..

[7] Werbowyj R.S., Gray D.G. (1984). Optical Properties of (Hydroxypropyl)cellulose Liquid Crystals. Cholesteric Pitch and Polymer Concentration. Macromolecules.

[8] Kosho H., Hiramatsu S., Nishi T., Tanaka Y., Kawauchi S., Watanabe J. (1999). Thermotropic Cholesteric Liquid Crystals in Ester Derivatives of Hydroxypropylcellulose. High Perform. Polym..

[9] Kawaguchi A., Aoki R., Hayata K., Furukawa M., Fukawa M., Furumi S. (2019). Fabrication of Human-Friendly Liquid Crystal Materials with α-Ionone. J. Photopolym. Sci. Technol..

[10] Saito S., Hayata K., Furumi S. (2020). Cholesteric Liquid Crystals from Cellulose Derivatives with Alkyl Ether Groups. J. Photopolym. Sci. Technol..

[11] Baba Y., Saito S., Iwata N., Furumi S. (2021). Synthesis and Optical Properties of Completely Etherified Hydroxypropyl Cellulose Derivatives. J. Photopolym. Sci. Technol..

[12] Yamagishi T., Sixou P. (1995). Preparation and Characteristics of Cholesteric Gel from Pentyl Ether of Hydroxypropyl Cellulose. Polymer.

[13] Suto S., Hasegawa S. (2002). Self-Colored Crosslinked Cholesteric Liquid Crystalline Solid Films of Hydroxypropyl Cellulose. J. Mater. Sci..

[14] Yamagishi T., Guittard F., Godinho M.H., Martins A.F., Cambon A., Sixou P. (1994). Comparison of Thermal and Cholesteric Mesophase Properties among the Three Kind of Hydroxypropylcellulose (HPC) Derivatives. Polym. Bull..

[15] De Vries H. (1951). Rotatory Power and Other Optical Properties of Certain Liquid Crystals. Acta Crystallogr..

[16] Furumi S. (2010). Recent Progress in Chiral Photonic Band-Gap Liquid Crystals for Laser Applications. Chem. Rec..

[17] Shimamura K., White J.L., Fellers J.F. (1981). Hydroxypropylcellulose, a Thermotropic Liquid Crystal: Characteristics and Structure Development in Continuous Extrusion and Melt Spinning. J. Appl. Polym. Sci..

[18] Onogi S., Asada T. (1980). Rheology and Rheo-Optics of Polymer Liquid Crystals. Rheology.

[19] Vshivkov S.A., Rusinova E.V., Saleh A.S.A. (2021). Rheological Properties of Liquid Crystalline Solutions of Cellulose Derivatives. Polym. Sci. Ser. A.

[20] Ureña-Benavides E.E., Ao G., Davis V.A., Kitchens C.L. (2011). Rheology and Phase Behavior of Lyotropic Cellulose Nanocrystal Suspensions. Macromolecules.

[21] Burghardt W.R. (1998). Molecular Orientation and Rheology in Sheared Lyotropic Liquid Crystalline Polymers. Macromol. Chem. Phys..

[22] Tseng S.L., Laivins G.V., Gray D.G. (1982). Propanoate Ester of (2-Hydroxypropyl)cellulose: A Thermotropic Cholesteric Polymer that Reflects Visible Light at Ambient Temperatures. Macromolecules.

[23] Ogiwara Y., Iwata N., Furumi S. (2021). Viscoelastic Properties of Cholesteric Liquid Crystals from Hydroxypropyl Cellulose Derivatives. J. Photopolym. Sci. Technol..

[24] Ogiwara Y., Iwata N., Furumi S. (2022). Unpublished results.

[25] Ho F.F.L., Kohler R.R., Ward G.A. (1972). Determination of Molar Substitution and Degree of Substitution of Hydroxypropyl Cellulose by Nuclear Magnetic Resonance Spectrometry. Anal. Chem..

[26] Hou H., Reuning A., Wendorff J.H., Greiner A. (2000). Tuning of the Pitch Height of Thermotropic Cellulose Esters. Macromol. Chem. Phys..

[27] Laivins G.V., Gray D.G. (1985). Optical Properties of (Acetoxypropyl)cellulose Mesophases: Factors Influencing the Cholesteric Pitch. Polymer.

[28] Ishizaki T., Uenuma S., Furumi S. (2015). Thermotropic Properties of Cholesteric Liquid Crystal from Hydroxypropyl Cellulose Mixed Esters. Kobunshi Ronbunshu.

